# Age-associated sex difference in the expression of mitochondria-based redox sensitive proteins and effect of pioglitazone in nonhuman primate brain

**DOI:** 10.1186/s13293-023-00551-6

**Published:** 2023-09-28

**Authors:** Sumit Jamwal, Jennifer K. Blackburn, John D. Elsworth

**Affiliations:** Department of Psychiatry, Yale University School of Medicine, New Haven, CT USA

**Keywords:** Pioglitazone, Uncoupling protein, Oxidative stress, Nonhuman primate, Striatum, Substantia nigra

## Abstract

**Background:**

Paraoxonase 2 (PON2) and neuronal uncoupling proteins (UCP4 and UCP5) possess antioxidant, anti-apoptotic activities and minimize accumulation of reactive oxygen species in mitochondria. While age and sex are risk factors for several disorders that are linked with oxidative stress, no study has explored the age- and sex-dependent expression of PON2 isoforms, UCP4 and UCP5 in primate brain or identified a drug to activate UCP4 and UCP5 in vivo. Preclinical studies suggest that the peroxisome proliferator-activated receptor gamma agonist, pioglitazone (PIO), can be neuroprotective, although the mechanism responsible is unclear. Our previous studies demonstrated that pioglitazone activates PON2 in primate brain and we hypothesized that pioglitazone also induces UCP4/5. This study was designed to elucidate the age- and sex-dependent expression of PON2 isoforms, UCP4 and UCP5, in addition to examining the impact of systemic PIO treatment on UCP4 and UCP5 expression in primate brain.

**Methods:**

Western blot technique was used to determine the age- and sex-dependent expression of UCP4 and UCP5 in substantia nigra and striatum of African green monkeys. In addition, we tested the impact of daily oral pioglitazone (5 mg/kg/day) or vehicle for 1 or 3 weeks on expression of UCP4 and UCP5 in substantia nigra and striatum in adult male monkeys. PIO levels in plasma and cerebrospinal fluid (CSF) were determined using LC–MS.

**Results:**

We found no sex-based difference in the expression of PON2 isoforms, UCP4 and UCP5 in striatum and substantia nigra of young monkeys. However, we discovered that adult female monkeys exhibit greater expression of PON2 isoforms than males in substantia nigra and striatum. Our data also revealed that adult male monkeys exhibit greater expression of UCP4 and UCP5 than females in substantia nigra but not in striatum. PIO increased UCP4 and UCP5 expression in substantia nigra and striatum at 1 week, but after 3 weeks of treatment this activation had subsided.

**Conclusions:**

Our findings demonstrate a sex-, age- and region-dependent profile to the expression of PON2, UCP4 and UCP5. These data establish a biochemical link between PPARγ, PON2, UCP4 and UCP5 in primate brain and demonstrate that PON2, UCP4 and UCP5 can be pharmacologically stimulated in vivo, revealing a novel mechanism for observed pioglitazone-induced neuroprotection. We anticipate that these outcomes will contribute to the development of novel neuroprotective treatments for Parkinson’s disease and other CNS disorders.

## Plain language summary

Parkinson’s disease (PD) is less common in women than men, which may be related to the protective effect of high levels of estrogens in women that maintain the activity of neuroprotective proteins in brain mitochondria. Our previous work suggests that paraoxonase-2 (PON2), uncoupling protein-4 (UCP4) and uncoupling protein-5 (UCP5) play vital roles in maintaining the health of brain dopamine neurons that are lost in PD. This work tested the hypothesis that female primate brains expresses higher levels of these proteins than males. In addition, this research investigated whether estrogen regulates the expression these factors and whether they can be pharmacologically activated later in life to protect dopamine neurons at a time when symptoms of PD typically emerge. The results indicate that before puberty when estrogen levels in females are relatively low, there is no difference in PON2, UCP4, UCP5 brain levels between males and females, but in adults PON2 is up to 3 × higher in females compared with males in regions relevant to PD, consistent with estrogen activation of PON2. Earlier studies have shown that pioglitazone can be neuroprotective in several adverse brain conditions, although the mechanism is not clear. The current research demonstrates that pioglitazone transiently activates by about twofold the expression of PON2, UCP4, UCP5 in vivo in primate brain, suggesting their involvement in the neuroprotective properties of the drug. Overall, the current data provides impetus for further work on activating protective factors that alter mitochondrial dynamics and function, leading to improved understanding and treatment of multiple diseases.

## Highlights

- PON2, UCP4 and UCP5 possess antioxidant, anti-apoptotic activities and minimize reactive oxygen species accumulation in mitochondria.
- Age-associated sex difference exists in expression of PON2 isoforms in adult STR and SN region of NHPs with higher levels found in females.
- Age-associated sex difference exists in expression of UCP4 and UCP5 in SN region of NHPs with higher levels found in males.
- Pioglitazone is first drug to induce in vivo expression of UCP4 and UCP5 in STR and SN regions.

## Introduction

Parkinson’s disease (PD) is an age-associated neurodegenerative disease characterized pathologically by the gradual loss of dopaminergic (DA) neurons in the substantia nigra (SN), which leads to the depletion of DA terminals in the striatum (STR) [52]. Although its etiopathogenesis is unknown in most cases, there is abundant evidence to implicate a key role of mitochondrial dysfunction and oxidative stress in PD [44]. The involvement of mitochondrial dysfunction in PD has been demonstrated by the reduced activity of mitochondrial complex I activity in SN of PD patients [47] and in animal models, where the parkinsonian-inducing toxins, MPTP and rotenone inhibit complex I and produce selective nigrostriatal DA neuron degeneration [28, 50]. A relatively high basal level of oxidative stress exists in SN, which is thought to contribute to the particular vulnerability of SN DA neurons in PD [19, 20, 39]. Current treatments for PD are limited to amelioration of symptoms [51] and treatment strategies that target the progressive course of PD are urgently needed. A potential approach to this is enhancement of endogenous neuroprotective systems and one promising target to combat mitochondrial dysfunction and oxidative stress is mitochondria-based antioxidant and antiapoptotic proteins, such as paraoxonase 2 (PON2) and uncoupling proteins (UCPs) [3, 26].

PON2 is an ubiquitously expressed enzyme located in mitochondria and endoplasmic reticulum that possess potent antioxidant, anti-apoptotic and anti-inflammatory activities [3, 9]. In in vitro PD models, overexpression of PON2 reduces reactive oxygen species (ROS), whereas its deficiency increases ROS [40]. Recently, PON2 deficiency in mice has been shown to be associated with motor deficits and impact DA-related genes that are important for survival of DA neurons, demonstrating a functional consequence of altered PON2 expression [10]. Our laboratory has demonstrated a distinct sex-bias in expression of PON2 in adolescent non-human primate (NHP) brain (mean age 3 years), with greater abundance occurring in females [23, 25]. In addition, we reported the existence of two PON2 isoforms (i.e., 39 kDa and 41 kDa) in multiple brain regions, with highest expression of both in striatum [23]. Like PON2, UCPs are transmembrane proteins present in the inner mitochondrial membrane, where they control the accumulation of ROS during oxidative phosphorylation [19, 20]. Currently, six homologs have been identified (UCP1–6) in mammals with UCP4 and UCP5 being distinguished by their predominant expression in central nervous system (CNS) neurons [37]. UCP4 and UCP5 work synergistically to maintain oxidative balance and ATP production [19, 20]. Thus, stimulating UCP4 and UCP5 expression is a potential approach to combat mitochondrial dysfunction and oxidative stress in PD [17, 26]. A paucity of data exists on UCP4 and UCP5 protein expression at different stages of life and distribution in brain, but emerging evidence supports an important role for them in protecting DA neurons from ROS. For example, UCP4 and UCP5 overexpression in SH-SY5Y cells results in better preservation of mitochondrial membrane potential, cellular ATP levels, and lower oxidative stress under conditions of MPP^+^-induced neurotoxicity [7, 27]. In addition, UCP4 and UCP5 are down-regulated in mice lacking DJ-1, a gene associated with an early onset form of PD [58]. Therefore, PON2, UCP4 and UCP5 are potential neuroprotective targets for PD [4, 26]. However, no study has explored whether the expression of PON2, UCP4 and UCP5 is dependent on age or sex in STR and SN, which influence risk and progression of PD [6, 18].

Recently, we found that the anti-diabetic drug pioglitazone (PIO), upregulates PON2 in male adult mice and NHP brain [3, 4]. PIO targets the transcription factor peroxisome proliferator-activated receptor gamma (PPARγ), which regulates genes involved in anti-inflammatory responses, mitochondrial biogenesis, and oxidative stress defense [24]. Thus, PON2 is a novel target of PIO and PPARγ, but it is not yet known whether PIO can induce expression of UCP4 and UCP5 in brain and this is addressed in the current study. Here, we use PIO as a pharmacological tool to stimulate the PPARγ pathway rather than to investigate its clinical utility.

The greater prevalence and incidence of PD in males compared to females [36, 38] has been at least partly attributed to a sex difference in susceptibility of nigrostriatal DA neurons to oxidative stress, with the female sex hormone estradiol thought to convey this protection [12, 33] and which may involve maintenance of higher basal expression of PON2 protein in females [13, 25]. In addition to an estradiol–PON2 interaction, it is not known whether estradiol’s neuroprotective effect is mediated through expression of UCP4 and UCP5, if so then there should be a male–female difference in their expression in adults but not in young animals before the age of puberty. This question is addressed in the current study. Since humans share much greater similarity with nonhuman primates (NHPs) than with rodents in terms of the endocrine system, organization of the central DA neurons and the pharmacological response to therapeutics [35, 42], the present study used NHPs to elucidate the age- and sex-dependent expression of the mitochondria-based neuroprotective proteins, PON2, UCP4 and UCP5, in the STR and SN, in addition to examining the impact of PIO treatment on UCP4 and UCP5 expression.

## Methods

African green monkeys (*Chlorocebus sabaeus*) were used in all studies. Animals were housed at the St. Kitts Biomedical Research Foundation, an AAALAC accredited facility. Studies were carried out in accordance with the “Guide for the Care and Use of Laboratory Animals”. All studies were approved by the IACUC. Euthanasia was carried out after injection of an overdose of pentobarbital followed by brain perfusion with cold saline, dissection, and freezing samples in liquid nitrogen. Tissue samples were shipped to New Haven, Connecticut in a cryogenic liquid nitrogen vapor shipper and then stored in a freezer set to maintain at -80 °C until analysis. Other tissues collected from animals in Experiments 1 and 2 have been and will be used in multiple other studies.

### Experiment 1: Sex bias in PON2, UCP4 and UCP5 expression in young and adult NHP brain

Young monkeys aged 11–28 days (female mean = 18.6, range 12–26; male mean = 18.2, range 11–28) and adult male monkeys (5–7 kg, mean estimated age 6–8 years) were used in this study.

### Experiment 2: Impact of PIO on UCP4 and UCP5 expression in adult male NHP’s brain

We analyzed tissue from an earlier study, where adult males monkeys were administered PIO orally at 5 mg/kg/day mixed with jam for 7 days (*n* = 5) or 21 days (n = 5). Control monkeys received jam only (vehicle control) for 7 days (*n* = 2) or 21 days (*n* = 3) [4]. The dose of PIO used in this study was based on the findings of Swanson et al. [53] who reported that repeated administration of 5 mg/kg oral PIO in rhesus monkeys reduced indices of DA neuron damage inflicted by the parkinsonian toxin, MPTP. The dose of PIO used in the current study and by Swanson et al. produced peak plasma levels in the therapeutic range, 1–2 µg/mL [4, 46, 49, 53].

### Total protein measurement

Tissue was sonicated in cold lysis buffer (Cell Signaling Technology, Danvers, MA) with cOmplete™ Protease Inhibitor Cocktail (Roche) and then centrifuged for 15 min at 8,000 × g at 4 °C. Total protein content in the supernatant was determined by the BCA assay (Pierce™ BCA protein assay kit, Thermo Scientific, Rockford, IL, USA).

### Western blot analysis

Western blot protocol was carried out using Bio-Rad (Hercules, CA) equipment and consumables for stain-free protein quantification, following the manufacturer's instructions. The supernatant was placed in 4 × Laemmli loading buffer followed by protein denaturation in a heating block for 5 min at 100 °C. Protein separation was performed on stain-free midi-Protean TGX gels before transfer to nitrocellulose membranes using the turbo transfer method and imaged with the ChemiDoc imaging system. Membranes were then blocked for 1 h at room temperature with 5% nonfat dry milk in Tris buffered saline wash buffer containing 0.1% Tween 20. Following this, membranes were incubated overnight in blocking buffer at 4 °C with primary antibody (anti-PON2 antibody (1:1000; ab183710, Abcam, Cambridge, MA), anti-UCP4 antibody (1:1000; ab183886, Abcam, Cambridge, MA), anti-UCP5 antibody (1:1000; ab221123, Abcam, Cambridge, MA). Membranes were washed and then incubated for 2 h at room temperature with anti-rabbit IgG, HRP-linked Antibody (1:10,000; #7074, Cell Signaling Technology, USA) in blocking buffer. After washing, the antibody complex was visualized by Clarity chemiluminescence (Bio-Rad Laboratories) and imaged with the ChemiDoc imaging system. PON2, UCP4 and UCP5 expression was normalized to total lane protein using Image Lab software (Bio-Rad Laboratories) within ChemiDoc XRS + (Bio-Rad Laboratories, Hercules, CA) [23, 25].

### Statistical analysis

Data are expressed as the mean ± SEM. The normality of each comparison group was assessed and confirmed by the Shapiro–Wilk test and homogeneity of variances was confirmed using the Brown–Forsythe test for homogeneity of variances. In Experiment 1, values from males and females in each region were compared by two-tailed unpaired Student’s *t* test. In Experiment 2, the effect of PIO treatment in each region was assessed by one-way ANOVA, followed by Tukey HSD post-hoc test for multiple comparisons using Prism 9 (GraphPad, La Jolla, CA). p < 0.05 was considered statistically significant in all analyses.

## Results

**Fig. 1:**
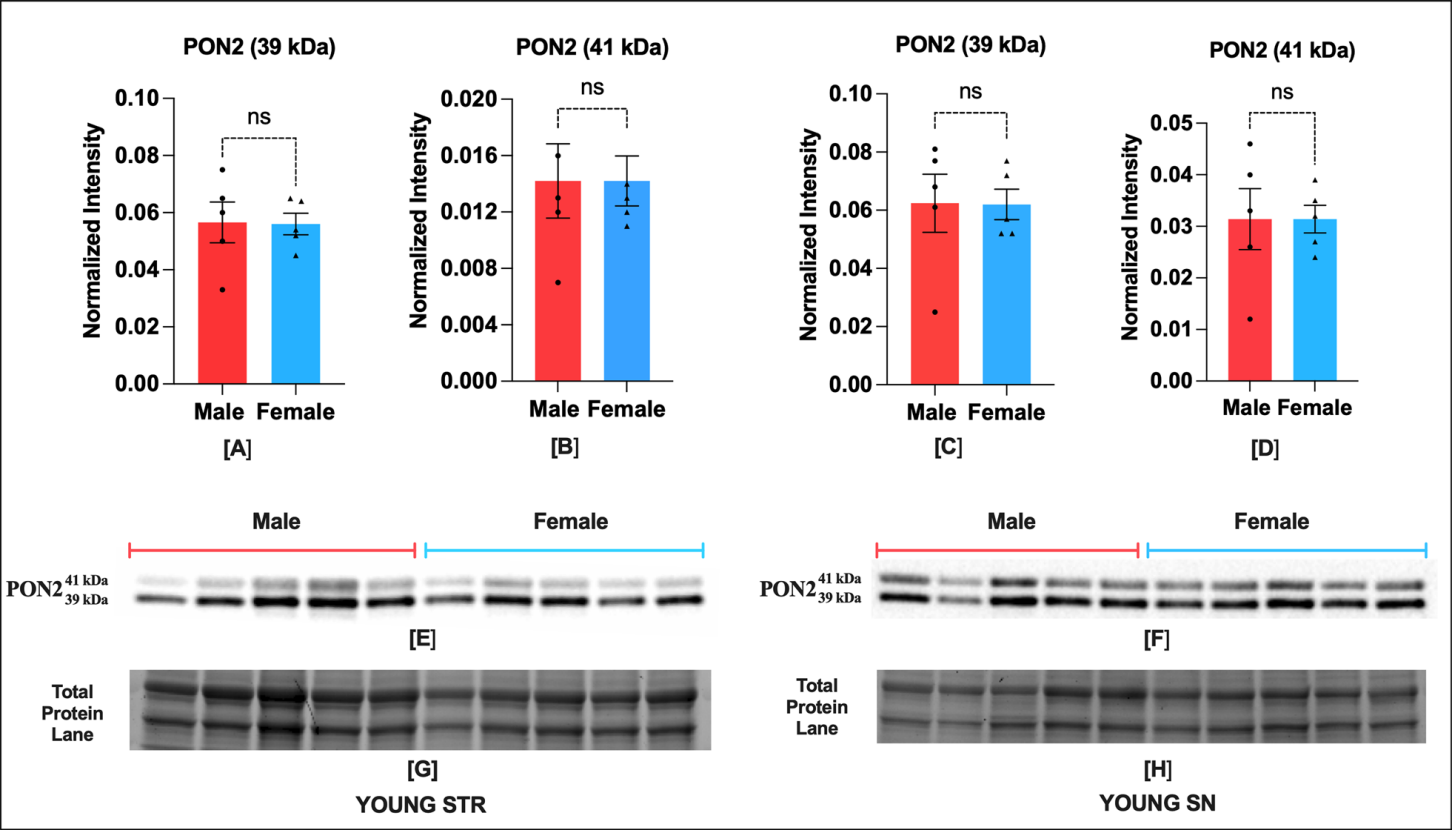
Young NHP brain has no sex bias in PON2 isoforms expression in STR and SN regions (*n* = 5). Protein expression of PON2 isoforms, i.e., 39 kDa and 41 kDa in STR (**A**, **B**) and SN (**C**, **D**), respectively. Representative blot showing PON2 isoforms expression in STR (**E**), and SN (**F**). Corresponding image of total protein in STR (**G**) and SN (**H**). Optical density of UCP5 and UCP4 bands were normalized to total protein per lane. The normality of infant male and female data was confirmed by a Shapiro–Wilk test. 39 kDa PON2 STR Male (W = 0.97, p = 0.89) Female (*W* = 0.91, *p* = 0.50); 41 kDa PON2 Male (*W* = 0.96, *p* = 0.87) Female (*W* = 0.80, *p* = 0.09); 39 kDa PON2 Male (*W* = 0.84, *p* = 0.18) Female (*W* = 0.83, *p* = 0.15); 41 kDa PON2 SN Male (*W* = 0.97, *p* = 0.87) Female (*W* = 0.97, *p* = 0.90). Data were expressed as mean ± SEM. Male and female data in each region was compared by two-tailed unpaired Student's *t* test. 39 kDa PON2 STR (two-tailed *t* (8) = 0.07, *p* = 0.94), 41 kDa PON2 STR (two-tailed *t* (8) = 0.0, *p* > 0.99), 39 kDa PON2 SN (two-tailed *t* (8) = 0.03, *p* = 0.97), 41 kDa PON2 SN (two-tailed *t* (8) = 0.0, *p* > 0.99)

**Fig. 2:**
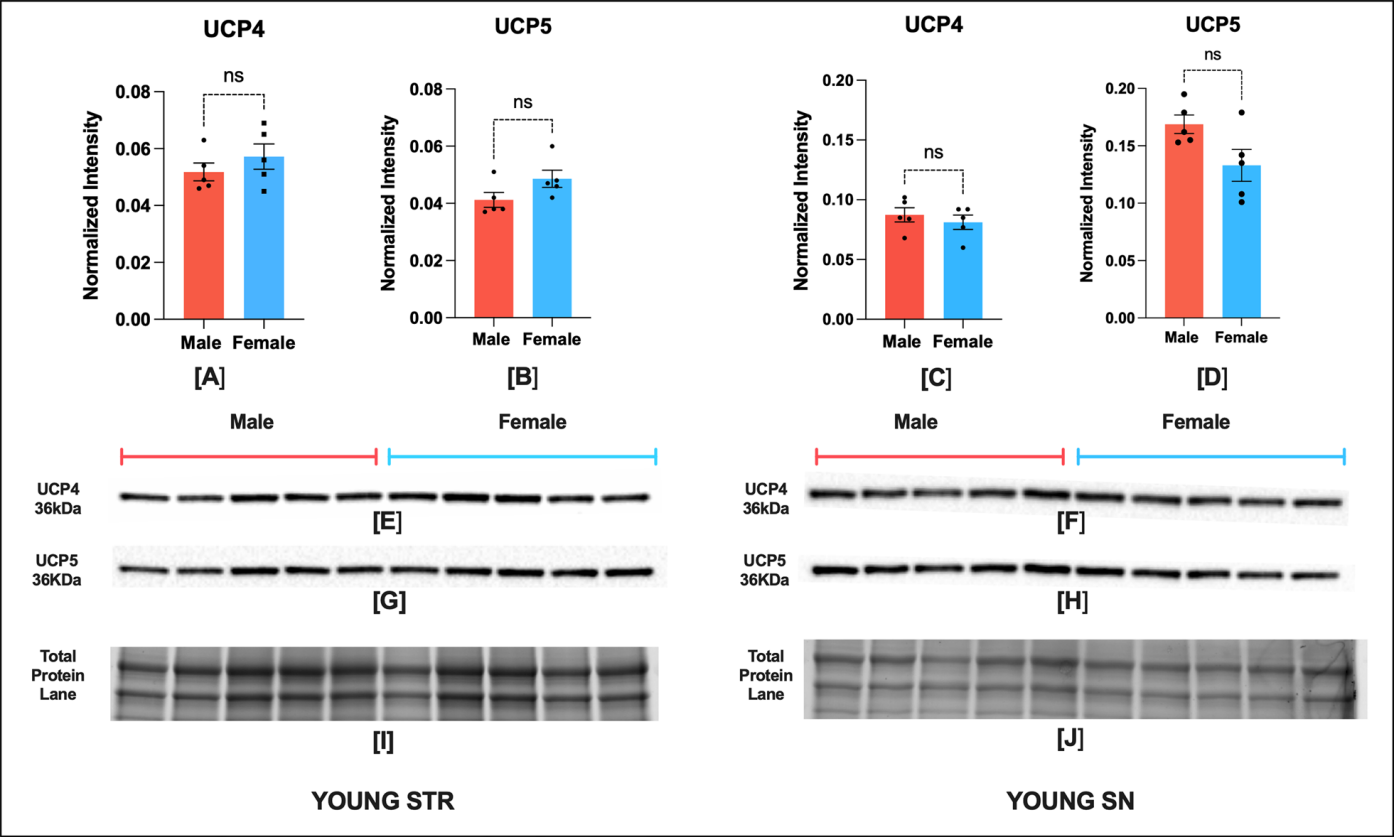
Young NHP brain has no sex bias in UCP4 and UCP5 expression in STR and SN regions (*n* = 5). Protein expression of UCP4 and UCP5 in STR (**A**, **B**) and SN (**C**, **D**), respectively. Representative blot showing UCP4 and UCP5 expression in STR (**E**, **G**), and SN (**F**, **H**) region. Corresponding image of total protein in STR (**I**) and SN (**J**). Optical density of UCP5 and UCP4 bands were normalized to total protein per lane. The normality of Infant male and female data was confirmed by a Shapiro–Wilk test. UCP4 STR Male (*W* = 0.86, *p* = 0.25) Female (*W* = 0.96, *p* = 0.81); UCP5 STR Male (*W* = 0.78, *p* = 0.057) Female (*W* = 0.84, *p* = 0.18); UCP4 SN Male (*W* = 0.93, *p* = 0.65) Female (*W* = 0.86, *p* = 0.25); UCP5 SN Male (*W* = 0.88, *p* = 0.35) Female (*W* = 0.93, *p* = 0.64). Data were expressed as mean ± SEM. Male and female data in each region was compared by two-tailed unpaired Student's *t* test. UCP4 STR (two-tailed *t* (8) = 0.99, *p* = 0.34), UCP5 STR (two-tailed *t* (8) = 1.85, *p* = 0.10), UCP4 SN (two-tailed *t* (8) = 0.73, *p* = 0.48), UCP5 SN (two-tailed *t* (8) = 2.23, *p* = 0.055)

**Fig. 3:**
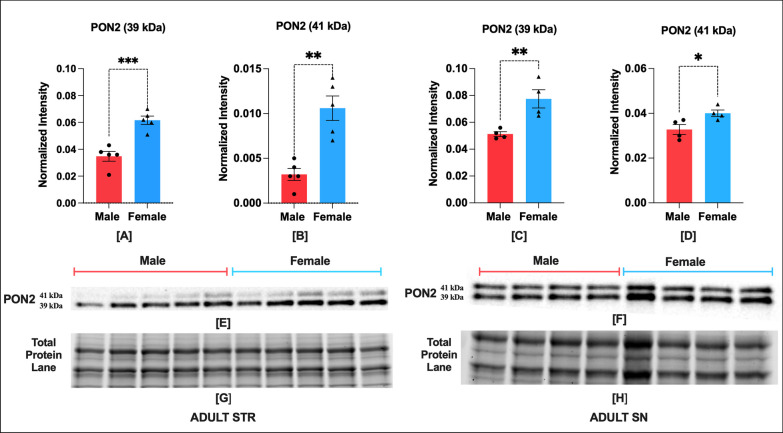
Adult NHP brain has sex bias in PON2 isoforms expression in STR and SN regions. STR (*n* = 5) and SN (*n* = 4). Protein expression of PON2 isoforms, i.e., 39 kDa and 41 kDa in STR (**A**, **B**) and SN (**C**, **D**), respectively. Representative blot showing PON2 isoforms expression in STR (**E**), and SN (**F**). Corresponding image of total protein in STR (**G**) and SN (**H**). Optical density of UCP5 and UCP4 bands were normalized to total protein per lane. The normality of infant male and female data was confirmed by a Shapiro–Wilk test. 39 kDa PON2 STR Male (*W* = 0.84, *p* = 0.17) Female (*W* = 0.97, *p* = 0.87); 41 kDa PON2 Male (*W* = 0.95, *p* = 0.77) Female (*W* = 0.92, *p* = 0.54); 39 kDa PON2 Male (*W* = 0.92, *p* = 0.58) Female (*W* = 0.91, *p* = 0.51); 41 kDa PON2 SN Male (*W* = 0.87, *p* = 0.33) Female (*W* = 0.95, *p* = 0.73). Data were expressed as mean ± SEM. Male and female data in each region was compared by two-tailed unpaired Student's *t* test. 39 kDa PON2 STR (two-tailed *t* (6) = 3.75, p = 0.0095), 41 kDa PON2 STR (two-tailed *t* (8) = 4.87, *p* = 0.0012), 39 kDa PON2 SN (two-tailed *t* (8) = 0.03, *p* = 0.97), 41 kDa PON2 SN (two-tailed t (6) = 2.72, p = 0.034). Asterisks indicate statistical significance in comparison with vehicle group, *p < 0.05, **p < 0.01, ***p < 0.001

**Fig. 4:**
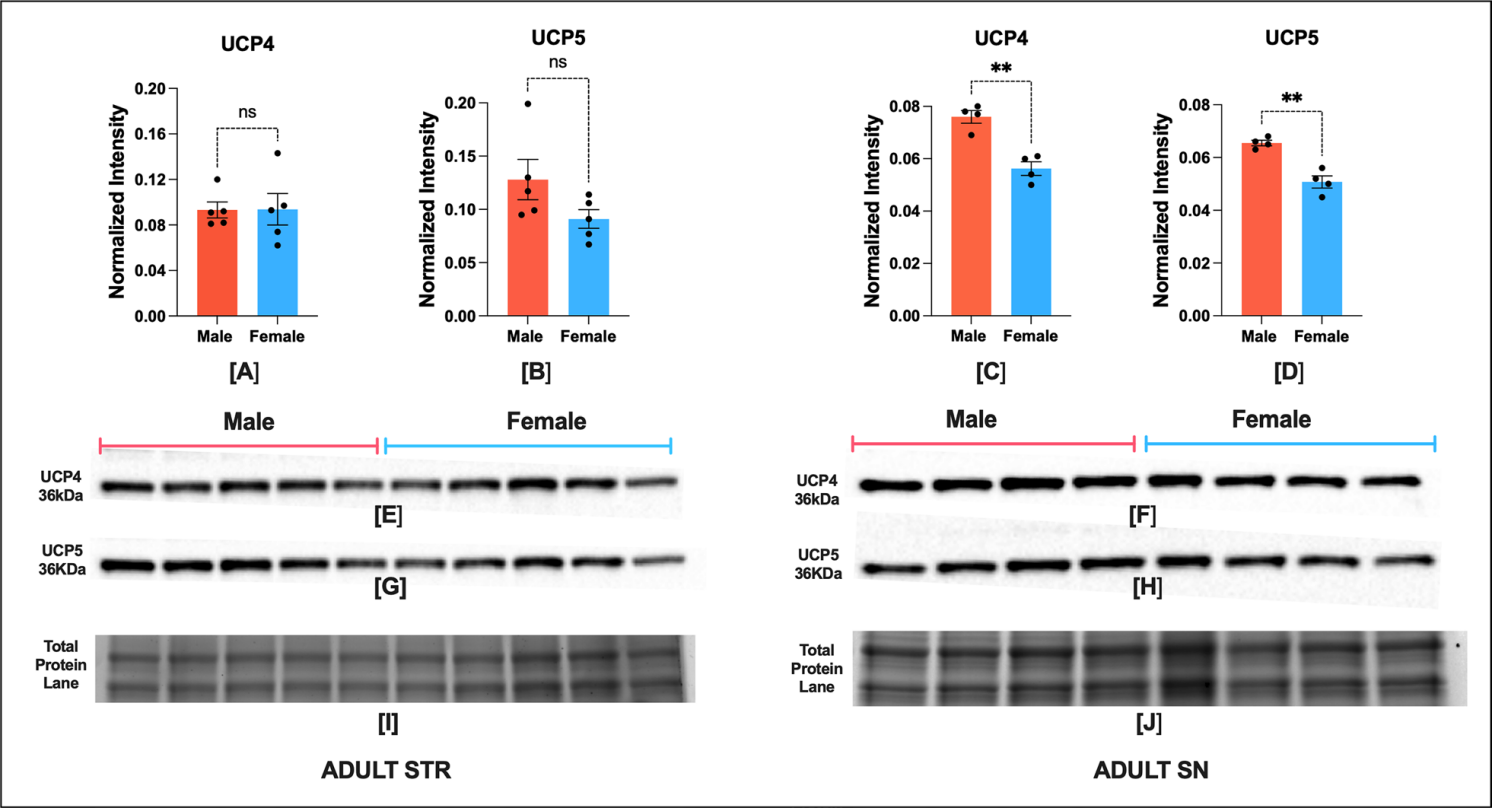
Adult NHP brain has sex bias in UCP-4 and UCP-5 expression in SN but not in STR region. STR (*n* = 5) and SN (*n* = 4). Protein expression of UCP4 and UCP5 in STR (**A**, **B**) and SN (**C**, **D**), respectively. Representative blot showing UCP4 and UCP5 expression in STR (**E**, **G**), and SN (F, H) region. Corresponding image of total protein in STR [I] and SN [J]. Optical density of UCP5 and UCP4 bands were normalized to total protein per lane. Optical density of UCP5 and UCP4 bands were normalized to total protein per lane. The normality of infant male and female data was confirmed by a Shapiro–Wilk test. UCP4 STR Male (*W* = 0.80, *p* = 0.085) Female (*W* = 0.91, *p* = 0.52); UCP5 STR Male (*W* = 0.82, *p* = 0.13) Female (*W* = 0.95, *p* = 0.79); UCP4 SN Male (*W* = 0.85, *p* = 0.23) Female (*W* = 0.90, *p* = 0.46); UCP5 SN Male (*W* = 0.99, *p* = 0.99) Female (*W* = 0.99, *p* = 0.97). Data were expressed as mean ± SEM. Male and female data in each region was compared by two-tailed unpaired Student's *t* test. UCP4 STR (two-tailed *t* (8) = 0.84, *p* = 0.93); UCP5 STR (two-tailed t (8) = 1.78, *p* = 0.11)**;** UCP4 SN (two-tailed *t* (6) = 5.57, *p* = 0.0014); UCP5 SN (two-tailed *t* (6) = 5.87, *p* = 0.0011). Asterisks indicate statistical significance in comparison with vehicle group, *p < 0.05, **p < 0.01

**Fig. 5:**
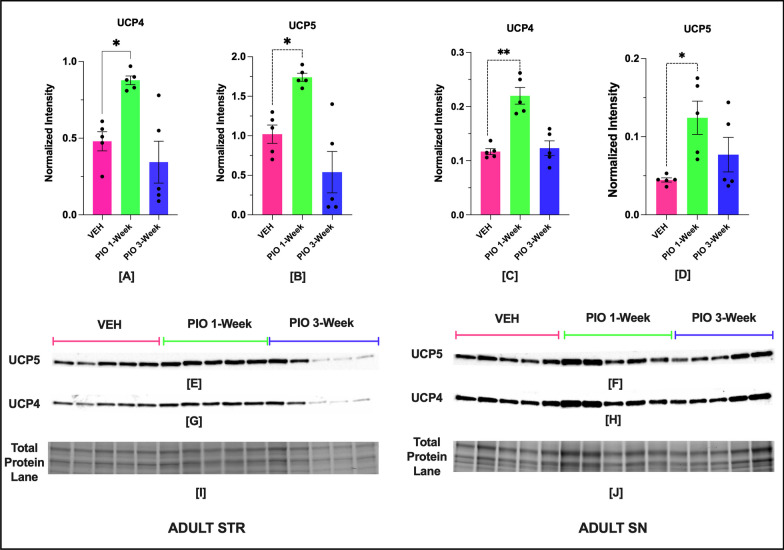
Impact of 1-week and 3-week PIO treatment on UCP-4 and UCP-5 protein expression in STR and SN of adult male NHPs (*n* = 5). Protein expression of UCP4 and UCP5 in STR (**A**, **B**) and SN (**C**, **D**), respectively. Representative blot showing UCP4 and UCP5 expression in STR (**E**, **G**), and SN (**F**, **H**) region. Corresponding image of total protein in STR (**I**) and SN (**J**). Optical density of UCP5 and UCP4 bands were normalized to total protein per lane. Optical density of UCP5 and UCP4 bands were normalized to total protein per lane. The normality of data in each group was confirmed by a Shapiro–Wilk test. UCP4 STR VEH (*W* = 0.88, *p* = 0.32), PIO 1 week (*W* = 0.95, *p* = 0.78), PIO 3 weeks (*W* = 0.84, *p* = 0.16); UCP5 STR VEH (*W* = 0.91, *p* = 0.51), PIO 1 week (*W* = 0.96, *p* = 0.81), PIO 3 weeks (*W* = 0.81, *p* = 0.10); UCP4 SN VEH (*W* = 0.87, *p* = 0.27), PIO 1 week (*W* = 0.87, *p* = 0.27), PIO 3 weeks (*W* = 0.93, *p* = 0.61); UCP5 SN VEH (*W* = 0.91, *p* = 0.51), PIO 1 week (*W* = 0.88, *p* = 0.34), PIO 3 weeks (*W* = 0.80, *p* = 0.08). Data were expressed as mean ± SEM. Data from different groups in each region were first analyzed for homogeneity of variance using Brown–Forsythe test (UCP4 STR: *F* (2, 12) = 1.551, *p* = 0.2518), UCP5 STR: *F* (2, 12) = 1.492, *p* = 0.2649); UCP4 SN: *F* (2, 12) = 1.593, *p* = 0.2435), UCP5 SN: *F* (2, 12) = 2.097, *p* = 0.1655)) and then compared by one-way ANOVA followed by Tukey HSD post-hoc test for multiple comparisons. UCP4 STR; one-way ANOVA: *F* (2, 12) = 9.91, *p* = 0.0029), UCP5 STR; one-way ANOVA: *F* (2, 12) = 12.94, *p* = 0.0010); UCP4 SN; one-way ANOVA: *F* (2, 12) = 22.47, *p* < 0.0001), UCP5 SN; one-way ANOVA: *F* (2, 12) = 5.06, *p* < 0.025). Asterisks indicate statistical significance in comparison with vehicle group, *p < 0.05, **p < 0.01

There is no sex difference in expression of PON2 isoforms (39 kDa and 41 kDa), UCP4 and UCP5 in both, STR and SN regions of young monkey brains **(**Figs. 1, 2**)**. In contrast, adult monkeys display a sex bias in expression of both PON2 isoforms, with greater levels (130–300%) of both isoforms existing in STR and SN regions of females compared with males **(**Fig. 3**)**. On the other hand, a sex bias in UCP4 and UCP5 expression was seen in adult SN, but not in adult STR, with greater levels (130–135%) of both proteins existing in SN region of males compared with females **(**Fig. 4**)**. In the pharmacological study, repeated PIO administration up-regulated expression of UCP4 and UCP5, but the effect was duration dependent. In STR and SN, UCP4 and UCP5 expression was 170–280% higher than vehicle controls following 1-week treatment but not different following 3-week treatment with PIO **(**Fig. 5**)**. This reduction in UCP expression at 3 weeks was not due to difference in PIO absorption or metabolism, as there was no change in plasma and CSF concentrations of PIO and its metabolites between the 1- and 3-week treatment groups [4].

## Discussion

The biggest risk factors for PD are age and sex, and there is not a clear understanding of the molecular basis for each of these determinants or how their effects might be mitigated. Compelling evidence has been accumulated to indicate that PON2, UCP4 and UCP5 play a vital role in managing redox signaling by regulating ROS accumulation [26, 40], and that these proteins are crucial for neuronal survival. The accumulated biochemical impact of aging, such as gradually diminishing mitochondrial function and the chronic exposure to higher basal level of oxidative stress in SN are thought to contribute to the selective vulnerability of DA neurons and onset of PD [55], with effects induced by estrogens considered to provide relative neuroprotection to females [33].

The observed higher expression of PON2 in STR and SN of healthy adult female monkeys is consistent with high circulating levels of estradiol in the blood. Healthy female mice possess a higher mitochondrial respiration and lower oxidative stress compared to males and these differences are suppressed by ovariectomy but not orchidectomy [11], pointing to a key role of estradiol in the beneficial oxidative stress balance in females. Female African green monkeys, similar to rhesus and cynomolgus monkeys, reach puberty at about 3 years of age and in captivity live as long as 30 years, with menopause occurring after the 20 years of age [2, 57]. The adult females in this study were cycling normally and estimated to be 6–8 years, based on a facility age estimation rubric, which was scored by a veterinarian during an evaluation of each animal’s behavior and physical condition. The mean estradiol level in adult female monkeys of reproductive age is at least ten times that in males [34]. Accordingly, it is reasonable to presume that gonadal hormones contributed to our finding of sex bias in PON2 isoforms expression in adult STR and SN that does not occur in young NHP.

We observed a greater expression of UCP4 and UCP5 under normal physiological conditions in SN of adult males compared with females, suggesting no effect of estradiol on UCP4 and UCP5 in this brain region. The literature on the influence of sex of UCP in rodents is mixed. For example, older female rats have higher UCP4 and UCP5 levels in mitochondria compared with similarly aged males [15, 16]. On the other hand, it is interesting to note that in male and female rats either gonadectomy or exogenous sex hormone (estradiol or dihydrotestosterone) treatment for 3–4 weeks does not affect the expression of UCP2, UCP4 or UCP5 in brain [43]. Consistent with this latter study, we interpret the observed greater expression of UCP4 and UCP5 in adult male SN as a compensatory response to the higher basal oxidative stress in adult males [20, 21, 39] that is not present early in life and which is not affected by gonadal hormones.

Extensive research during past few decades has identified PPARs as essential players involved in the control of PON2 [4, 5, 14, 31]. Recently, our research group demonstrated that the PPARγ ligand, PIO, upregulates PON2 in mouse and NHP brain [3, 4]. UCP gene transcription [48, 56] is also regulated by PPARs, yet the current study is the first to identify an interaction between PPARγ ligands and UCP4 or UCP5. However, a previous study did document that PIO activates UCP2 mRNA expression in mouse skeletal muscle [48]. Earlier studies by us and others explored the role of UCP2 in brain under physiological and pathophysiological conditions, typically by measuring uncoupling activity or UCP2 mRNA, as detection of UCP2 protein in brain has proved challenging due to its uneven distribution and the historical inadequacy of reliable anti-UCP2 antibodies [1, 8, 21, 22]. Based on a study using stem cells before and after differentiation to neurons, another explanation for the difficulty in detecting UCP2 protein in brain is that UCP2 is preferentially expressed in cells with high proliferative potential, whereas UCP4 is strongly associated with non-proliferative highly differentiated neuronal cells [45]. Consequently, in terms of protecting DA neurons, a shift in focus from UCP2 to UCP4 and UCP5 as targets for PD therapeutics seems appropriate.

Interestingly, it has been demonstrated that UCP4 and UCP5 are downstream effectors in the established NF-κB c-Rel pro-survival pathway [19, 32]. For example, NF-κB c-Rel dimers are involved in initiating neuroprotective signals and neuronal resistance to stressful conditions by inducing the expression of UCP-4, UCP5 and antiapoptotic genes, such as MnSOD and Bcl-xL [30]. Furthermore, in cultured rat cortical neurons PIO upregulates expression of anti-apoptotic factor Bcl-xL and mRNA of NF-κB c-Rel [29]. Consistent with a link between DA neuron integrity and the NF-κB/UCP pathway, NF-κB/c-Rel deficiency caused PD-like symptoms with progressive pathology in mice [41].

## Perspectives and significance

Previous evidence indicates that PON2, UCP4 and UCP5 serve as mitochondrial surveillance factors that mitigate the effects of oxidative stress; however, there is limited understanding of their endogenous regulation and there were no pharmacological tools to enhance their expression. We report novel findings that (1) an age-associated sex difference exists in expression of PON2 isoforms (i.e., 39 kDa and 41 kDa) in adult STR and SN region of NHPs with higher levels found in females (2) an age-associated sex difference exists in expression of UCP4 and UCP5 in SN region of NHPs with higher levels found in males and (3) PIO is first drug to induce in vivo expression of UCP4 and UCP5 in STR and SN regions. Interestingly, PIO activation of UCP4 and UCP5 is transient, similar to our previous findings on expression of PON2 following PIO administration [4]. The waning of UCP4 and UCP5 activation after 3 weeks of daily PIO treatment is evidently due to existence of homeostatic mechanisms that preclude long-term escalations in their expression under physiological conditions. Future studies are now warranted to investigate the extent and persistence of PIO-induced neuronal UCP4 and UCP5 expression in adult female monkeys, and in PD models, in addition to defining the involvement of NF-κB/c-Rel pathway in PIO-mediated upregulation of UCP4 and UCP5. The outcomes of such investigations should provide clues to realizing the potential of UCPs as neuroprotective targets and lead to strategies to regulate their activity. Overall, the current data should provide impetus for further work on activating protective factors that have potential to alter mitochondrial dynamics and function leading to improved understanding and treatment of multiple diseases [54].

## Data Availability

The data sets used and/or analysed during the current study are available from the corresponding author on reasonable request.

## Abbreviations

CSF: Cerebrospinal fluid
CNS: Central nervous system
DA: Dopaminergic
MPTP: 1-Methyl-4-phenyl-1,2,3,6-tetrahydropyridine
NHP: Nonhuman primate
PIO: Pioglitazone
PON2: Paraoxonase 2
PPARγ: Peroxisome proliferator-activated receptor gamma
ROS: Reactive oxygen species
SN: Substantia nigra
STR: Striatum
UCP: Uncoupling protein
